# Studies in Austral Bryaceae (Bryopsida). III. A Preliminary Account with Keys to *Rosulabryum* J.R. Spence in Chile

**DOI:** 10.3897/phytokeys.185.70407

**Published:** 2021-11-15

**Authors:** John R. Spence

**Affiliations:** 1 California Academy of Sciences, Department of Botany, 55 Music Concourse Drive, Golden Gate Park, San Francisco, USA Department of Botany, California Academy of Sciences San Francisco United States of America

**Keywords:** Bryaceae, Chile, mosses, *Rosulabryum*

## Abstract

A preliminary study of the genus *Rosulabryum* J.R. Spence in Chile is presented, with brief species descriptions, notes on ecology and distributions, and a taxonomic key. The following 12 species are confirmed with vouchered specimens; *Rosulabryumandicola* (Hook.) Ochyra, *Rosulabryumbillarderii* (Schwägr.) J.R. Spence, *Rosulabryumcampylothecium* (Taylor) J.R. Spence, *Rosulabryumcapillare* (Hedw.) J.R. Spence, *Rosulabryumcoloratum* (Müll. Hal.) J.R. Spence, *Rosulabryumdensifolium* (Brid.) Ochyra, *Rosulabryumlongidens* (Thér.) J.R. Spence, *Rosulabryummacrophyllum* (Cardot & Broth.) Ochyra, *Rosulabryumperlimbatum* (Cardot) Ochyra, *Rosulabryumpuconense* (Herzog & Thér.) J.R. Spence, *Rosulabryumrubens* (Mitt.) J.R. Spence, and *Rosulabryumtorquescens* (Bruch *ex* De Not.) J.R. Spence. *Rosulabryumcanariense* (Brid.) Ochyra is tentatively excluded as the Chilean material can be referred to *R.coloratum*. Similarly, *Rosulabryumviridescens* (Welw. & Duby) Ochyra is tentatively excluded since the Chilean plants do not match the African type, but instead appear to be atypical plants of *R.campylothecium*.

## Introduction

With a known moss flora of more than 900 species, Chile has a rich but incompletely documented bryoflora (Müller 2009). Recent studies involving herbarium collections and field work (Larraín 2016; Ireland et al. 2017; Drapela and Larraín 2020; Larraín et al. 2020) suggest that many more species will be discovered. The country has a great diversity of climates, geological substrates, and vegetation communities, ranging from tropical arid deserts in the north, montane and alpine climates along the Andean Mountain chain, Mediterranean-climate regions in the center of the country, to temperate and subantarctic forests and moorlands in the south.

The Bryaceae is a large world-wide family that occurs in a wide variety of habitats and climates and can represent as much as 10% of the species richness in local and regional moss floras. Chile has an extremely rich and incompletely known Bryaceae flora, with an estimated 85 species, based on revisions and recent collecting (Ochi 1980, 1982; Müller 2009; Ireland et al. 2017; Spence 2020a). These field studies have also revealed species that appear to be new to science, mostly in the genera G*emmabryum* J.R. Spence & H.P. Ramsay, *Ochiobryum* J.R. Spence & H.P. Ramsay, and *Plagiobryoides* J.R. Spence.

One of the most easily recognized and common genera is *Rosulabryum* J.R. Spence, which is well represented in the southern hemisphere. The genus currently consists of ca. 75 described species (J. Spence unpublished data), with 12 well documented species in Chile. However, much of the country remains under collected, especially in the north and in the Andes, thus it is likely that additional species will be found with more intensive field work. Of particular note is the presence of a diverse montane *Rosulabryum* flora in the northern and central Andes which may extend southward into the Chilean Andes.

*Rosulabryum* occurs in a clade with BrachymeniumSchwägr.sect.Brachymenium, sister to a clade consisting of *Plagiobryum* Lindb., *Plagiobryoides* J.R. Spence and *Ptychostomum* Hornsch. (Pedersen et al. 2006). The molecular studies that include more than one species in the genus include Cox and Hedderson (2003), Pedersen et al. (2003) and Pedersen et al. (2006). These studies show a well-supported *Rosulabryum* clade using several different species. However, the relationships and phylogenetic position of the *Rosulabryum* and the related *Ptychostomum* clades remain unresolved at this time.

## Method and materials

Collections were obtained from various herbaria, primarily CONC, MO and NY. Additional field work in the Tierra del Fuego region of Chile (Magellanes Province) has been completed and collections are currently being studied in the author’s personal herbaria. Much of the recent floristic work has been done in the southern regions of Bío Bío, Aisén and Magallanes (e.g., Larraín 2016; Ireland et al. 2017; Drapela and Larraín 2020; Spence 2020a; unpublished data). Nomenclature for the species documented in this study follows Spence (2020b). This study is a preliminary treatment of the genus for Chile to help guide identification of collections and field work.

## Results

Of the 12 species documented in this study, their distributions include four main elements, 1) widespread temperate, 2) southern temperate, 3) subantarctic/*Nothofagus*, and 4) Neotropical montane. The widespread element includes *R.capillare*, *R.rubens* and *R.torquescens*. Southern temperate species include *R.billarderii*, *R.campylothecium*, and *R.macrophyllum*, with the first two extending to Australasia. The subantarctic element is represented by *R.perlimbatum*, which extends to southern New Zealand. The Neotropical montane element includes *R.andicola* and *R.densifolium*. An endemic element may also exist, as currently *R.longidens* and *R.puconense* are endemic to Chile, with potentially one record of the former reported from Argentina which needs to be verified. *Rosulabryumcoloratum* has an anomalous distribution, as it occurs in montane regions of Bolivia as well as lower elevations of south-central Chile. In general, these distributions conform at least in part to well documented and similar distributions among the vascular plant flora of Chile (Moreira-Muñoz 2011). Overall, the diversity of *Rosulabryum* in Chile is similar to that in other well studied regions, including Australasia (14 spp.) and North America (16 spp.), but appears to be more diverse than the western Palearctic (8 spp.).

*Rosulabryum* is characterized by a combination of predominantly gametophyte characters, including mostly rosulate habit to sometimes evenly foliate stems, ovate to obovate or spathulate leaves, a well-developed limbidium, serrulate, serrate or denticulate distal leaf margins, costa with well-developed stereid band, nodding capsule, well-developed peristome with appendiculate cilia, small spores, rhizoidal tubers, and filiform gemmae in the leaf axils (Mohamed 1979; Spence 1996). In terms of gametophyte morphology, *Rosulabryum* is most similar to *Rhodobryum*. *Rhodobryum* differs in producing distinctive stolons, a weakly developed to nearly absent stereid band in the costa, and a lack of rhizoidal tubers and leaf axil gemmae. Both genera often have polysetose perichaetia, but this feature is much more common in *Rhodobryum*. In addition, the chromosomes of *Rhodobryum* are significantly different in structure not only from *Rosulabryum* but the rest of the family (cf. Ramsay and Spence 1996).

### Key to *Rosulabryum* of Chile

**Table:** 

1	Filiform gemmae present, usually in axils of sterile shoot leaves	**2**
1’	Gemmae absent	**3**
2	Plants large, at least some leaves > 3 mm, distal margins sharply serrate; costa of innovations short-excurrent; predominantly terricolous	***Rosulabryumandicola* (in part)**
2’	Plants small, leaves < 2 (2.5) mm, distal margins serrulate, costa of innovation leaves long excurrent; terricolous or lignicolous-epiphytic, often on tree trunks and large branches	***Rosulabryumlongidens***
3	Plants small, leaves mostly < 2 (2.5) mm, spirally twisted around stem when dry, costa excurrent in long ± straight awn	***Rosulabryumcapillare***
3’	Plants small to robust, if spirally twisted around stem then leaves > 3 mm or costa short-excurrent	**4**
4	Plants small, leaves < 2 mm, distal leaf margins smooth to serrulate, stems rosulate to somewhat evenly foliate, leaves often with reddish tints; rhizoidal tubers red to orange or red-brown, cell walls protuberant	**5**
4’	Plants medium to large, leaves mostly > 2.5mm, distal leaf margins finely serrulate to sharply serrate or denticulate, stems variable but often rosulate, leaves mostly green to yellow-green, rarely somewhat reddish-tinged, rhizoidal tuber color various, cells smooth, lacking protuberant walls	**6**
5	Sexual condition dioicous; costa excurrent in medium-length awn, limbidium distinct, leaf margins serrulate, rhizoidal tubers red, cell walls strongly protuberant, diameter to ca. 260 (280) µm; capsules 2–3 mm long	***Rosulabryumrubens***
5’	Sexual condition synoicous or polyoicous with single sex shoots, costa percurrent to short excurrent in short awn, limbidium weak, sometimes absent distally, margins smooth to finely serrulate; rhizoidal tubers red-brown, red to orange, cell walls weakly protuberant, diameter to 600 µm; capsules 4–6 mm long	***Rosulabryumpuconense***
6	Limbidium weak distally, of 1 row or sometimes nearly absent; leaves concave; distal laminal cells firm-walled to often incrassate	**7**
6’	Limbidium distinct distally, usually of 2 or more rows; leaves flat or weakly concave; distal laminal cells thin to firm-walled but usually not incrassate	**10**
7	Plants in tight rosulate tufts when dry, leaves imbricate, golden-yellow or yellow-green; costa excurrent in long ± straight awn; rhizoidal tubers lacking	***Rosulabryumcampylothecium***
7’	Plants in loose rosulate to comal tufts or stems evenly foliate, leaves somewhat imbricate to shrunken or twisted when dry, colors various but not golden-yellow; costa excurrent in short often recurved awn; tubers present	**8**
8’	Plants ± evenly foliate; leaves spirally twisted around stem when dry; leaf apex acute, costa excurrent in short recurved awn; tubers brown to red-brown	***Rosulabryumcoloratum***
8	Plants in one or more interrupted rosulate tufts; leaves weakly imbricate to irregularly contorted when dry; leaf apex broadly acute to rounded or obtuse; costa excurrent in short recurved or straight awn; tubers red, scarlet, brown or yellow-brown	**9**
9	Sexual condition autoicous or rarely synoicous; leaf broadly acute at tip, costa tapering towards leaf tip, leaves not strongly keeled; rhizoidal tubers bright red to scarlet	***Rosulabryumcanariense***
9’	Sexual condition dioicous or rhizo-autoicous; leaf rounded-obtuse at tip, costa very thick to apex, leaves strongly keeled; rhizoidal tubers brown to yellow-brown	***Rosulabryumviridescens***
10	Sexual condition synoicous or rarely autoicous; plants medium-sized, leaves 2–3 mm, obovate, costa excurrent in medium-length awn; rhizoidal tubers scarlet to red	***Rosulabryumtorquescens***
10’	Sexual condition dioicous or rhizo-autoicous; plants medium to robust, leaves mostly > 3 mm, ovate, obovate to spathulate, costa percurrent to excurrent in short awn; rhizoidal tubers dark red, brown to red-brown	**11**
11	Limbidium very wide, (3) 6–8 rows, often imparting whitish-hyaline border to leaf	**12**
11’	Limbidium narrower, (2) 3–4 rows, colored to clear, not imparting whitish-hyaline border to leaf	**13**
12	Leaves spirally twisted around stem when dry, distal margins sharply serrate; limbidium 3–4 rows; filiform leaf axil gemmae usually present	***Rosulabryumandicola* (in part)**
12	Leaves irregularly contorted to somewhat imbricate when dry, distal margins serrulate; limbidium 4–8 rows; filiform leaf gemmae absent	***Rosulabryumperlimbatum***
13	Plants elongate evenly foliate, to 8–10 cm, leaves narrowly ovate, distal margins sharply serrate to denticulate, teeth often double	***Rosulabryumdensifolium***
13’	Plants rosulate, not evenly foliate, mostly < 2 cm long, leaves obovate to broadly ovate, distal margins serrulate to singly serrate	**14**
14	Leaves ovate to oblong, ±imbricate when dry; distal margins serrulate	***Rosulabryummacrophyllum***
14’	Leaves obovate to spathulate, irregularly contorted when dry; distal margins sharply serrate	***Rosulabryumbillarderii***

### Species accounts

#### 1. Rosulabryum andicola

Taxon classificationPlantaeBryalesBryaceae

(Hook.) Ochyra

B3FA6C4A-9ECF-5676-A810-22579E69C6DB

##### Remarks.

A widespread African-Neotropical species found from Chile north to the Southwestern US. This species replaces *R.billarderii* in warmer climates in Chile and is rare south of the Maule Region. It is found principally on damp to dry soil or soil over rock, rarely on fallen logs and tree stumps. The species is characterized by dioicous sexual condition, strongly rosulate stems, large leaves that are spirally twisted around the stem when dry, a costa excurrent in a short awn, a very well-developed wide limbidium, sharply serrate distal leaf margins, and brown filiform gemmae in the leaf axils of sterile shoots. The rhizoidal tubers are red to red-brown and can be > 1 mm in diameter. (Illustrations: Mohamed 1979: 422, as *Bryumandicola*; Magill 1987: 382, as *B.andicola*).

**Representative specimen examined**: Region VIII, Bío Bío Province, Ecological Reserve Coligual, 37°23'S 71°40'W, ca. 630 m, on forest floor, clay banks along road with adjacent native forest, *R.R. Ireland & G. Bellolio 35234*, 21 Nov. 2002 (MO).

#### 2. Rosulabryum billarderii

Taxon classificationPlantaeBryalesBryaceae

(Schwägr.) J.R. Spence

C96399A2-32AD-5D34-BD72-34C7FAC05F7A

##### Remarks.

The common widespread species of large *Rosulabryum* from central Chile south to Aisén. The species is also found in Argentina, the Falkland Islands, Australia, New Zealand and Macquarie Island (Spence and Ramsay 2019). In the Magellanes Region it is largely replaced by *R.perlimbatum*. The species occurs in a variety of forested and semi-open habitats, often in shade on mesic to damp soil banks, fallen logs, tree stumps and soil-covered ledges. It is characterized by dioicous sexual condition, strongly rosulate stems, large leaves that are irregularly contorted when dry, a costa excurrent into a short awn, a strong limbidium, serrate leaf margins, and brown to red-brown rhizoidal tubers (Illustrations: Mohamed 1979: 407, as *Bryumbillarderii*; Spence and Ramsay 2006: 335, as *R.billarderii*; Fife 2015: 84, as *R.billarderii*).

##### Representative specimens examined.

Aisén Region, Provincia Capitán Prat, cruzando pasarela Río Ñadis, en bosque de *Nothofagusdombeyi*, 47°29'49"S, 72°56'51"W, alt. ca. 70 m, sobre tronco caído en claro del bosque, *J. Larraín & R. Vargas 26799*, 19 Ene 2007 (CONC): Region VIII, Bío Bío Province, road from Tomeco to Florida, 2 km N from Hwy 0–50, 36°57'S 72°40'W, alt. ca. 190 m, on soil bank, R.R. Ireland & G. Bellolio 32401 (MO).

#### 3. Rosulabryum campylothecium

Taxon classificationPlantaeBryalesBryaceae

(Taylor) J.R. Spence

13954154-3DCE-5A95-B05C-9ABDE0EBBAD5

##### Remarks.

A common temperate species in the central and south regions of Chile from Coquimbo Region to at least the Los Lagos Region but absent from colder subantarctic climates. It also occurs in Australia and New Zealand (Spence and Ramsay 2019). The species is found on exposed to partially shaded damp to drying soil, sand, and rock ledges, often in open woodlands or on cliffs and outcrops. The species is characterized by dioicous sexual condition, medium-sized imbricate golden to yellow-green strongly concave leaves, a costa excurrent in a long often denticulate awn, a weak or nearly absent limbidium distally, serrate distal leaf margins, and the absence of rhizoidal tubers (Illustrations: Spence and Ramsay 2006: 335, as *R.campylothecium*; Fife 2015: 85, as *R.campylothecium*).

##### Representative specimens examined.

Region VIII, Prov. Ñuble, Cobquecura, rock cliffs near ocean, 36°05'S 72°48'W, alt. ca. 0 m, on cliff, *R.R. Ireland & G. Bellolio 32374*, 9 Oct 2001 (MO): Region VIII, Prov. Concepción, Puda Beach, 36°29'S 72°54'W, alt. ca. 0 m, *R.R. Ireland & G. Bellolio 32267*, 7 Oct. 2001 (MO).

#### 4. Rosulabryum capillare

Taxon classificationPlantaeBryalesBryaceae

(Hedw.) J.R. Spence

C0D54655-EE4B-580C-A81A-B79A552431AC

##### Remarks.

A worldwide temperate species found in a wide variety of habitats, but most often on damp shaded soil or fallen logs and tree stumps. Its distribution in Chile remains poorly understood but it is common in the more temperate southern regions such as Bío Bío (Ireland et al. 2017). It is characterized by dioicous sexual condition, rosulate stems, small obovate leaves that are spirally twisted around the stem when dry, a costa excurrent into a medium to long awn, moderately well-developed limbidium, serrate distal leaf margins, and small brown rhizoidal tubers (Illustrations: Syed 1973: 270 as *Bryumcapillare*; Spence and Ramsay 2006: 336, as *R.capillare*; Hallingbäck et al. 2008: 349, as *B.capillare*; Fife 2015: 86, as *R.capillare*; Lüth 2019: 923 as *Ptychostomumcapillare*; Holyoak 2021: 72, as *P.capillare*).

##### Representative specimens examined.

Region VIII, Prov. Concepción, Bellavista Creek, San José Farm, 36°41'S 72°56'W, alt. ca. 90 m, on rotten log, stream with adjacent native forest with predominantly *Nothofagus*, *Aetoxiconpunctatum* & *Peumusboldus*, *R.R. Ireland & G. Bellolio 33903*, 23 Nov. 2001 (MO): Region VIII, Bío Bío Province, Saltillo del Itata, small falls on Itata River, 37°04'S 72°09'W, ca. 210 m, on brick wall beside poplar trees by river, *R.R. Ireland & G. Bellolio 34950*, 28 Oct. 2002 (MO).

#### 5. Rosulabryum coloratum

Taxon classificationPlantaeBryalesBryaceae

(Müll. Hal.) J.R. Spence

CBD5DF7A-C91B-5426-A96F-1C0CFFE37DD3

##### Remarks.

A relatively uncommon species found principally in semi-arid regions in the central and northern regions of Chile and adjacent areas of Bolivia, found on sandy soil or soil over rock, often near streams. The species is characterized by its dioicous sexual condition, evenly foliate stems, large narrowly ovate leaves that are spirally twisted around the stem when dry, a weak limbidium distally, and brown to red-brown rhizoidal tubers. Ochi (1980) had synonymized it with *R.canariense* but it is treated here as a good species (see below under *R.canariense*). (Illustration: Ochi 1980: 148, as *Bryumcanariense*).

##### Representative specimens examined.

Aisén Region, Provincia Capitán Prat, río Baker, pasando balsa Baker y luego camino río abajo, en bosque en cerro detrás de la casa de don Delmiro, junto a riachuelo, 47°15'46"S, 72°42'54"W, alt. ca. 100 m, en el suelo junto al arroyo, *J. Larraín & R. Vargas 26697*, 18 Ene 2007 (CONC); Region VIII, Prov. Ñuble, road from Cobquecura to Quiríhue, 24 km SE of Cobquecura, Mengel Creek, 36°13'S 72°36'W, alt. ca. 360 m, on sandy soil beside creek, *R.R. Ireland & G. Bellolio 32323*, 8 Oct 2001 (MO).

#### 6. Rosulabryum densifolium

Taxon classificationPlantaeBryalesBryaceae

(Brid.) Ochyra

C0BB0BFF-3527-5877-9028-A00AE3BB4268

##### Remarks.

A robust species distributed throughout the Neotropics, primarily along the mountain chains from Mexico south to Chile, and throughout the Andes (Ochi 1980). It is common at higher elevations, often above 3000 m, but can also occur in the lowlands, and is found along streams and in wetland areas on wet soil or rock. The species is characterized by its dioicous sexual condition, long evenly foliate stems with large narrowly ovate leaves, a costa excurrent into a medium to long awn, strong limbidium, sharply serrate to denticulate distal leaf margins, with the teeth often double, and brown to red-brown rhizoidal tubers (Ochi 1967: 30, as *Bryumdensifolium*).

##### Representative specimen examined.

Bío Bío Region, Prov. Ñuble, Renegado River at Aserradero bridge, 36°54'S 71°28'W, alt. ca. 1000 m, on muddy soil bank beside river, *R.R. Ireland & G. Bellolio 30831*, 9 Dec 2002 (MO).

#### 7. Rosulabryum longidens

Taxon classificationPlantaeBryalesBryaceae

(Thér.) J.R. Spence

13942ED4-56AD-5537-8337-189DAE35B465

##### Remarks.

A common species in the central regions of Chile, from the Bío Bío north to at least Coquimbo, usually on tree trunks and larger branches, stumps and fallen trees in forests, occasionally on soil. The species is characterized by dioicous sexual condition, small leaves, rosulate fertile stems, with numerous rosulate to evenly foliate innovations with leaves spirally twisted around the stem when dry, strong excurrent costa in medium to long awn which is sometimes red, brown to red-brown filiform gemmae in the leaf axils of sterile shoots, and small red to red-brown rhizoidal tubers. Ochi (1982) synonymized this with the Neotropical *R.pseudocapillare* (Besch.) Ochyra, but *R.longidens* is quite distinct from that species. The single report from Argentina (Ochi 1982; as *R.pseudocapillare*) needs to be re-examined as it may be a different species, thus *R.longidens* may be a Chilean endemic. It is the only epiphytic species in the genus in Chile. There are no known illustrations of this species.

##### Representative specimen examined.

Region VIII, Bío Bío Province, Lago Falls as south side of Laja River, 37°12'S 72°22'W, alt. ca. 170 m, on tree stump, *R.R. Ireland & G. Bellolio 34815*, 23 Oct. 2002 (MO); Region VIII, Prov. Arauco, Huillinco Falls, 37°45'S 73°22'W, alt. ca. 130 m, on base of large *Nothofagus* sp., falls and rock cliff in native forests, *R.R. Ireland & G. Bellolio 33662*, 12 Nov. 2001 (MO).

#### 8. Rosulabryum macrophyllum

Taxon classificationPlantaeBryalesBryaceae

(Cardot & Broth.) Ochyra

0B51940B-7357-5552-B882-C00E35612536

##### Remarks.

An uncommon species of moist to wet soil in the subantarctic moorlands and *Nothofagus* forests in southern Chile, reaching north to the Aisén Region (Ochi 1982; Larraín 2016)). The species is characterized by dioicous sexual condition, rosulate stems, large ovate leaves that are ±imbricate when dry, costa excurrent into a short awn, a fairly well developed limbidium, finely serrulate distal leaf margins, and brown to red-brown rhizoidal tubers. The species is also known from the Falkland Islands and Argentina (Illustrations: Ochi 1967: 33, as *Bryummacrophyllum*).

##### Representative specimen examined.

Región de Aisén, Provincia de Aisén, Comuna de Cisnes Parque Nacional Queulat, sector Angostura Risopatrón, sendero Los Colonos, bosque de *Amomyrtusluma*-*Nothofagus nitida-Laureliopsis philippiana*, sobre tronco podrido en la orilla del lago, 44°14'04"S, 72°30'24"W, elev. 130 m, *J. Larraín 43684*, *with R. Vargas*, *E. Muñoz & J.F. Croxatto*, 14 dic 2019 (CONC).

#### 9. Rosulabryum perlimbatum

Taxon classificationPlantaeBryalesBryaceae

(Cardot) Ochyra

0A162A85-47CC-520F-9AA4-5B0A121A1175

##### Remarks.

A common and widespread species of the subantarctic forests and moorlands of the Magallanes and Aisén Regions on damp to wet soil, fallen logs, and tree stumps (Ochi 1982). Reports from further north are likely misidentifications and are either *R.andicola* or *R.billarderii*. This is a robust species with dioicous sexual condition, rosulate stems, sometimes with 2+ interrupted clumps along stems, large leaves that are contorted to somewhat imbricate when dry, serrulate distal leaf margins, an extremely wide limbidium of 4+ rows distally, and brown to red-brown rhizoidal tubers. The wide limbidium often gives the leaves a white-margined appearance. The species is also found in Argentina, the Falkland Islands, and extreme southern New Zealand (Illustrations: Mohamed 1979: 422, as *Bryumperlimbatum*; Fife 2015: 87, as *R.perlimbatum*).

##### Representative specimen examined.

Prov. Antártica Chilena, Comuna Cabo de Hornos, Parque Nacional Alberto de Agostini, Isla Hoste, Península Dumas, Bahía Ibáñez, Caleta Yekadahby, 55°03'47“S, 68°25'19“W, on soil in shade in moist *Nothofagusbetuloides*-*N.pumilio*-*Maytenusmagellanica* forest on E-facing slope with extensive rock outcrops, *J.R. Spence 6059*, 15 January 2013(CAS).

#### 10. Rosulabryum puconense

Taxon classificationPlantaeBryalesBryaceae

(Herzog & Thér.) J.R. Spence

6977A47F-B391-5982-A4F0-F0EE2FA35CCB

##### Remarks.

This species was synonymized under *R.capillare* by Ochi (1980). However, it differs in significant ways. It is characterized by small rosulate to evenly foliate stems, small leaves that are somewhat imbricate to irregularly contorted when dry, costa excurrent in a short awn, a weak to sometimes absent limbidium, and smooth to finely serrulate distal leaf margins. Perhaps most striking are its sexual condition and rhizoidal tubers. Most collections are synoicous, but some are single-sex and thus would be considered dioicous. The species likely has the same sexual systems as *R.torquescens* and can be described as polyoicous. The rhizoidal tubers are similar to those produced by *R.rubens*, with often protuberant cell walls, but they are much larger. Some tubers are up to 600 µm across, with colors varying from red-brown to red or orange. *R.puconense* seems to have a preference for mesic to dry soil under shrubs and is especially common along the coasts from central Chile south to the Tierra del Fuego region. Although currently a Chilean endemic, it may ultimately be found in southern Argentina. There are no known illustrations of this species.

##### Representative specimen examined.

Prov. Antártica Chilena, Comuna Cabo de Hornos, Isla Grande de la Tierra del Fuego, Bahía Yendegaia, NNE shore opposite Caleta Ferrari, 54°50'28“S, 68°47'52“W, on dry exposed soil over rock outcrops near ocean, *J.R. Spence 6042*, 13 January 2013 (CAS): Region VIII, Bío Bío Province, Saltillo del Itata, small falls on Itata River, 37°04'S 72°09'W, ca. 210 m, on soil bank among poplar trees by river, *R.R. Ireland & G. Bellolio 34940*, 28 Oct. 2002 (MO).

#### 11. Rosulabryum rubens

Taxon classificationPlantaeBryalesBryaceae

(Mitt.) J.R. Spence

61DB58F9-C7BF-53EA-8891-0BE9CF12A072

##### Remarks.

A rare species generally found in disturbed habitats, especially on disturbed soil and concrete, and possibly introduced from the northern hemisphere. There is only one record from Chile in disturbed habitats in the capitol Santiago (Ochi and Mahu 1988, at HO, not seen!). It is characterized by its dioicous sexual condition, rosulate to somewhat evenly foliate stems, small leaves that are irregularly contorted when dry, costa excurrent into a medium-length awn, a narrow but usually distinct limbidium, and serrulate distal leaf margins. The plants are often reddish-tinged. The rhizoidal tubers are diagnostic; predominantly red, with strongly protuberant cell walls, from 100–280 µm in diameter, often arising from lower leaf axils or at the base of the stem on short rhizoids. The species has a scattered world-wide temperate distribution (Illustrations: Crundwell and Nyholm 1964: 630, as *Bryumrubens*; Lüth 2019: 938 as *Ptychostomumrubens*; Holyoak 2021: 232, as *P.rubens*).

#### 12. Rosulabryum torquescens

Taxon classificationPlantaeBryalesBryaceae

(Bruch ex De Not.) J.R. Spence

EEE29438-552F-5584-ACDB-7AF0637A0ED9

##### Remarks.

A widespread warm temperate to subtropical species in the northern hemisphere, Africa, Australasia and South America (Spence and Ramsay 2019). Although there are few collections from Chile, the species is likely to be common in the Mediterranean-climate regions of the country as far south as Los Lagos. It is found on soil, soil over rock and occasionally wood, including burnt wood, generally in exposed areas, often along road cuts. It is characterized by synoicous or rarely autoicous sexual condition, rosulate stems, medium-sized leaves that are irregularly contorted when dry, a costa excurrent in a medium to long awn, strong limbidium, serrate distal leaf margins, and scarlet, bright red to orange rhizoidal tubers (Illustrations: Syed 1973, 308 as *Bryumtorquescens*; Spence and Ramsay 2006: 347, as *R.torquescens*; Hallingbäck et al. 2008: 350, as *B.torquescens*; Lüth 2019: 939 as *Ptychostomumtorquescens*; Holyoak 2021: 257, as *P.torquescens*).

##### Representative specimens examined.

Los Lagos Region, Chiloé, communa de Ancud, Estación Biológica Senda Darwin, Al fondo de al “ciudad de los Muertos”, Sobre tronco caido ed bosque Quemado, 41°52'S 73°39'W, 300 m, *J. Larraín 23424*, 2 Feb. 2003 (CONC): Region VIII, Prov. Concepcion, Park “Jorge Alessandri” (Compania Manufacturera de Papeles y Cartones), on soil in clearing, 36°56'S 73°09'W, ca. 200–490 m, *R.R. Ireland & G. Bellolio 32819*, 19 Oct. 2001 (MO).

### Excluded species

*Rosulabryumcanariense* (Brid.) Ochyra. Ochi (1980) placed *R.coloratum* into synonymy under this species. Here they are treated as specifically distinct (see above under *R.coloratum*). True *R.canariense* is autoicous or synoicous, has interrupted rosulate tufts along the stem, irregularly contorted obovate leaves, and bright red, scarlet or orange rhizoidal tubers. I have not seen any material from Chile in collections examined in this study with these characters, thus it is tentatively excluded from South America, but is retained in the key pending additional field work. It is a northern hemisphere Mediterranean-climate species, also reported from South Africa, although the plants there are somewhat distinct from northern hemisphere plants.

*Rosulabryumviridescens* (Welw. & Duby) Ochyra. Ochi (1977) reported this species based on the type of *Bryumhamatum* Dusén, an illegitimate name as there was no formal description. More recently, I have identified several collections from the Bío Bío Region as this species. However, having recently obtained material of *R.viridescens* from South Africa, it is clear that the South American material is not that species (cf. Magill 1987). Most of the recently named collections are atypical specimens of *R.campylothecium*, while the *B.hamatum* type may be an aberrant form of *R.macrophyllum* or *R.perlimbatum* characterized by imbricate ovate leaves. Thus *R.viridescens* in tentatively excluded from Chile and South America, although it is retained in the key.

## Supplementary Material

XML Treatment for Rosulabryum andicola

XML Treatment for Rosulabryum billarderii

XML Treatment for Rosulabryum campylothecium

XML Treatment for Rosulabryum capillare

XML Treatment for Rosulabryum coloratum

XML Treatment for Rosulabryum densifolium

XML Treatment for Rosulabryum longidens

XML Treatment for Rosulabryum macrophyllum

XML Treatment for Rosulabryum perlimbatum

XML Treatment for Rosulabryum puconense

XML Treatment for Rosulabryum rubens

XML Treatment for Rosulabryum torquescens
